# A detailed dataset of motor insurance policies with coverage-specific financial information

**DOI:** 10.1016/j.dib.2026.112883

**Published:** 2026-05-23

**Authors:** Priscila Espinosa, Josep Lledó, David Atance

**Affiliations:** a Departamento de Economía Aplicada. Universitat de València. Avenida de los naranjos s/n., 46022 Valencia, Spain; b Departmento de Economía y Dirección de Empresas. Universidad de Alcalá. Plaza San Diego, s/n., 28801 Madrid, Spain

**Keywords:** Motor insurance, Non-life insurance, Risk, Pricing, Actuarial

## Abstract

This article presents a dataset of anonymised motor insurance policies from a Spanish insurance company covering the period 2022–2024. The dataset contains 354,140 policy-year observations and >185,000 unique insured policies per year, with a total of 47 variables. The dataset includes detailed information on policy and insured characteristics, driver and vehicle characteristics, as well as financial information such as premiums, exposure and claims. A systematic data processing and validation procedure was applied to ensure internal consistency, including the removal of duplicate policy-year records, standardisation of categorical variables and validation of numerical ranges. A key feature of the dataset is the detailed disaggregation of premiums, exposures, claim counts and incurred amounts by insurance coverage, including third-party liability (material and bodily injury), property damage, theft, fire, glass, legal protection and occupants. This structure supports reuse in actuarial pricing, risk modelling and insurance research.

Specifications Table

**Table:** 

Subject	Social Sciences
Specific subject area	*Insurance; Finance and Banking; Risk analysis; Non-life insurance*
Type of data	*Table (Excel file)*
Data collection	The data were collected from the internal information systems of a Spanish insurance company and correspond to anonymised microdata from motor insurance policies over a three-year period. The raw data were processed and normalised using R scripts, applying quality controls, range validation and internal consistency checks across variables.
Data source location	*Country: Spain.*
Data accessibility	Repository name: Mendeley Data Data identification number: 10.17632/sw4jmdb2sm.1 Direct URL to data: https://doi.org/10.17632/sw4jmdb2sm.1
Related research article	*None*

## 1 Value of the Data

• This dataset provides anonymised motor insurance microdata with a high level of detail, a type of information that is rarely accessible to the scientific community due to confidentiality and competitive constraints in the insurance sector. Each row represents information about the policyholder and the characteristics of their insured vehicle.
• A distinctive feature is the availability of financial information such as premiums, exposures, claims and incurred amounts, disaggregated by coverage rather than only at the aggregate policy level, allowing coverage-specific analyses of pricing adequacy, risk heterogeneity and loss dynamics.
• It enables policy-level analysis of risk behaviour, including claim occurrence and frequency, contract dynamics, and the relationship between driver, vehicle and policy characteristics.
• The data are well suited for methodological and applied research in actuarial pricing, risk analysis and portfolio segmentation, including comparisons across different driver profiles.

## 2 Background

The availability of high-quality datasets is a key element of empirical research across the social sciences. Several studies have released data resources in fields such as climatology [1], health [[2], [3]] and social sciences [4], improving transparency, reproducibility and empirical comparability.

In contrast, the insurance sector remains largely opaque. Access to detailed microdata is limited by confidentiality, competition and data protection constraints, restricting replication and cross-study comparison. Some datasets have nevertheless been published for life insurance [5], health insurance [6] and motor insurance [[7], [8], [9], [10]]. Spanish motor insurance datasets have long been used in actuarial research for claim prediction and pricing [[11], [12], [13]], however these data are not publicly available due to insurer confidentiality and privacy protections. A coverage-level breakdown is provided solely by [9], only for claims and for a limited share (15%) of the portfolio.

This data article presents a new anonymised motor insurance dataset from a Spanish insurer covering 2022–2024. Its main contribution is the detailed disaggregation of both premiums and claims by coverage, together with information on policies, drivers, vehicles, claims and contract dynamics. The article documents data collection, cleaning and structure to support transparency, replication and reuse.

## 3 Data Description

This article describes an anonymised dataset on motor insurance policies from a Spanish insurance company, covering the period from 1 January 2022 to 31 December 2024. The dataset accompanying this article is provided in spreadsheet format and is also hosted in https://doi.org/10.17632/sw4jmdb2sm.1. It contains a total of 354,140 observations and 47 variables. Each observation corresponds to an insured individual for a given year, and each variable represents a specific policy, vehicle, driver, claims or contractual characteristic.

In aggregate terms, the dataset includes more than 185,000 unique insured policies per year across the three years analysed, with 67,172, 118,835 and 168,133 policies in 2022, 2023 and 2024, respectively. The longitudinal structure allows the same insured individual to appear in multiple years, enabling the analysis of policy dynamics over time.

Table 1, Table 2, Table 3, Table 4 provide a detailed description of the 47 variables included in the dataset, organised into four groups according to their characteristics. Table 1 reports policy and insured identification variables, Table 2 describes variables related to driver and vehicle characteristics, Table 3 includes economic variables capturing key financial indicators related to policy premiums and exposure and Table 4 summarises risk exposure and claims information. For each variable, the corresponding definition and data type are reported.

**Table 1:** Description of variables. Group: Policy and insured characteristics.

Variable	Description	Group
***insured_id***	Unique identifier assigned to each policy number. This assigns a sequential ID based on the first appearance of each policy number. The policy number is only repeated when more than one year appears in the portfolio	Policy and insured characteristics
***Year***	Calendar year (2022, 2023 and 2024)	Policy and insured characteristics
***policy_type***	Categorical variable describing the main coverage structure of the motor insurance policy. The original detailed combinations are grouped into broader categories that are more meaningful from an actuarial perspective: - TP: basic third-party liability coverage only - TPG: third-party liability with glass coverage - CC: third-party liability combined with two or more additional coverages, such as theft, fire, total loss or glass - COMP_E: comprehensive with excess - COMP_N: comprehensive without excess	Policy and insured characteristics
***policy_status***	Indicates whether the policy was active (A) or cancelled (C) at calendar year.	Policy and insured characteristics
***business_type***	Classification of the policy according to its business origin: - NB: new_business - P: portfolio	Policy and insured characteristics
***payment_frequency***	Indicates the policy’s billing frequency: - A: annual - S: semiannual - Q: quarterly	Policy and insured characteristics
***bonus_score***	Indicator of the policyholder’s past claims experience: - G: positive reflects a favourable claims history - N: indicates a neutral profile - B: negative denotes a poor claims history	Policy and insured characteristics

**Table 2:** Description of variables. Group: Driver and vehicle characteristics.

Variable	Description	Group
***driver_age***	Age of the main driver associated with the policy, measured in years.	Driver and vehicle characteristics
***vehicle_age***	Age of the insured vehicle, also measured in years.	Driver and vehicle characteristics
***age_driving_licence***	Year in which the insured obtained the driving licence. Note: this variable records the calendar year of licence issuance and does not measure the length of time the licence has been held.	Driver and vehicle characteristics
***fuel_type***	Indicates the vehicle’s fuel type. In this dataset, only two categories are kept: - D: diesel - G: gasoline	Driver and vehicle characteristics
***vehicle_value***	Declared insured value of the vehicle.	Driver and vehicle characteristics
***seats***	Number of seats available in the insured vehicle.	Driver and vehicle characteristics
***power_to_weight_ratio***	Weight-to-power ratio of the vehicle (kg per horsepower), used as a proxy for performance characteristics.	Driver and vehicle characteristics
***vehicle_brand***	Brand of the insured vehicle as reported in the policy data.	Driver and vehicle characteristics
***municipality_type***	Geographic classification of the policyholder’s municipality: - I: inland - C: coastal - IS: islands	Driver and vehicle characteristics
***circulation_area***	Indicates the predominant circulation environment of the vehicle: - U: urban - R: rural	Driver and vehicle characteristics

**Table 3:** Description of variables. Group: Premiums.

Variable	Description	Group
***total_premium***	Total premium of the policy, calculated as the sum of all individual coverage premiums.	Premiums
***liability_premium***	Premium charged for third-party liability coverage.	Premiums
***property_damage_premium***	Premium corresponding to property damage coverage.	Premiums
***theft_premium***	Premium associated with theft coverage.	Premiums
***fire_premium***	Premium for fire coverage.	Premiums
***glass_premium***	Premium for glass coverage.	Premiums
***legal_protection_premium***	Premium for legal protection coverage.	Premiums
***occupants_premium***	Premium for personal-injury coverage of vehicle occupants.	Premiums

**Table 4:** Description of variables. Group: Exposure, claims and incurred.

Variable	Description	Group
***total_claims***	Indicates the total number of claims reported for the policy during the exposure period. Typical values range from 0 (no claims) to several claims per contract.	Exposure, claims and incurred
***liability_claims***	Total number of liability claims reported under the policy.	Exposure, claims and incurred
***liability_property_claims***	Number of liability claims related to material or property damage.	Exposure, claims and incurred
***liability_injury_claims***	Number of liability claims involving bodily injury.	Exposure, claims and incurred
***property_claims***	Count of property damage claims.	Exposure, claims and incurred
***theft_claims***	Number of theft-related claims.	Exposure, claims and incurred
***fire_claims***	Count of fire-related claims.	Exposure, claims and incurred
***glass_claims***	Number of glass-related claims.	Exposure, claims and incurred
***legal_protection_claims***	Number of legal protection claims.	Exposure, claims and incurred
***occupants_claims***	Number of claims under this coverage.	Exposure, claims and incurred
***total_incurred***	Represents the total incurred cost of claims for the policy, including paid amounts and outstanding reserves. Values are typically zero for policies without losses and positive when claims exist.	Exposure, claims and incurred
***liability_incurred***	Total incurred cost (paid + reserved) for all liability claims.	Exposure, claims and incurred
***liability_property_incurred***	Total incurred cost for liability claims involving material or property damage.	Exposure, claims and incurred
***liability_injury_incurred***	Total incurred cost for liability claims involving bodily injury.	Exposure, claims and incurred
***property_incurred***	Total incurred cost for property damage claims.	Exposure, claims and incurred
***theft_incurred***	Total incurred cost for theft claims.	Exposure, claims and incurred
***fire_incurred***	Total incurred cost for fire claims.	Exposure, claims and incurred
***glass_incurred***	Total incurred cost for glass claims.	Exposure, claims and incurred
***legal_protection_incurred***	Total incurred cost for legal protection claims.	Exposure, claims and incurred
***occupants_incurred***	Total incurred cost under this coverage.	Exposure, claims and incurred
***total_exposure***	Represents the effective time-on-risk for each policy, expressed in policy years. Values range from 0 to 1 for partial-year exposure.	Exposure, claims and incurred
***liability_exposure***	Amount of exposure associated with liability coverage.	Exposure, claims and incurred

•**(i) Policy and insured characteristics**

The first group of variables is designed to uniquely identify each record in the dataset. Each insured individual is assigned a unique numeric identifier (insured_id) that remains constant across the analysed years, which are indicated by the variable year (YYYY) taking the values 2022, 2023 or 2024. Although the insurance company provided its own internal identification codes, these were recoded by the authors to ensure internal consistency and to preserve data confidentiality. Not all policies are observed for the full three-year period, as some insureds leave (lapse) the portfolio while new ones join the portfolio during the sample period. Table 1 reports the variables included in the set of policy and insured characteristics.

The variable policy_type refers to the main insurance coverage structure. Original detailed combinations were grouped into broader, actuarially meaningful categories: *TP* (*third party*), basic third-party liability coverage; *TPG* (*third party with glass*), third-party liability including glass coverage; *CC* (*combined cover*), third-party coverage combined with two or more additional coverages such as theft, fire, total loss or glass; *COMP_E* comprehensive coverage with an excess; and *COMP_N* comprehensive coverage without an excess.

The variable policy_status indicates whether the policy is active (*A*) or cancelled (*C*) during the corresponding calendar year. The variable business_type distinguishes between existing portfolio policies (*P*) and new business (*NB*). The variable payment_frequency reports the billing frequency: annual (*A*), semiannual (*S*) or quarterly (*Q*). Finally, bonus_score is an indicator of the insured’s claims history, capturing the overall loss experience of the policyholder. The variable is classified into three categories: good (*G*), indicating a favourable claims history; neutral (*N*), representing an intermediate profile; and bad (*B*), reflecting an unfavourable claims history.

•**(ii) Driver and vehicle characteristics**

The second group of variables provides key information on the characteristics of the driver and the vehicle, from both the policyholder’s and the insurer’s perspective. These variables allow the analysis of the insured’s profile, coverage conditions, contracted product types, vehicle characteristics, usual area of circulation and the geographical classification of the municipality in which the vehicle is insured. Table 2 reports the variables included in the set of driver and vehicle characteristics.

The variable driver_age measures the age of the habitual driver, expressed in years and defined as an integer variable. The variable vehicle_age refers to the age of the insured vehicle, also measured in years and recorded as an integer. Similarly, age_driving_licence is an integer variable measuring the number of years since the insured obtained their driving licence. Fuel type (coded as fuel_type) is a categorical variable taking the values *D* (diesel), *G* (gasoline) or *NA* when the information is not available. The variable vehicle_value records the value of the vehicle as declared by the insured and also takes the value *NA* when this information is not available. The indicator power_to_weight_ratio captures vehicle performance in relation to its weight and is defined as the approximate weight-to-power ratio of the vehicle (kilograms per horsepower), used as a proxy for performance characteristics. The variable vehicle_brand identifies the brand of the insured vehicle as recorded in the policy.

The variable municipality_type categorizes the municipality in which the vehicle is operated and assumes one of the following values: *I* (interior), *C* (coastal) or *IS* (islands). Finally, the variable circulation_area identifies the habitual driving area in which the vehicle is predominantly operated: urban use (*U*), when it is mainly driven in cities or large municipalities; rural use (*R*), when it is used in small municipalities or rural areas; any other value is coded as *NA* (not available).

•**(iii) Premiums**

The third group of variables, displayed in Table 3, contains economic and financial information related to the price of the insurance policy. Premiums represent the net amount paid by insured individuals for their policy during the corresponding year and constitute a primary source of revenue for the insurance company. Premium levels vary according to the characteristics of the contracted insurance, such as coverage structure, the insured’s risk profile and policy conditions. Premium information is essential for the computation of key financial indicators, including loss ratios, portfolio sustainability and the profitability of different insurance products.

This group includes premiums associated with each contracted coverage: liability_premium, property_damage_premium, theft_premium, fire_premium, glass_premium, legal_protection_premium and occupants_premium. All these variables are recorded as numeric values. Zero may be observed for some coverages, reflecting the absence of the corresponding coverage in the insured’s policy. Finally, the variable total_premium is included and is defined as the sum of all individual coverage premiums.

•**(iv) Exposure, claims and incurred amounts**

The group of exposure, claims and incurred amount variables, as shown in Table 4, constitutes the analytical core for studying policy risk behaviour. The variable total_claims reports the total number of claims declared during the exposure period of the policy, taking the value zero when no claims occur and positive values when one or more events are recorded.

Within this point, a detailed breakdown of third-party liability coverage is provided. The variable liability_claims reports the total number of liability claims. Liability coverage is further disaggregated by the nature of damage. The variables liability_property_claims and liability_injury_claims record, the number of claims related to property (material) damage and bodily injury, respectively.

Beyond liability, the dataset includes information on other specific motor insurance coverages. For own damage coverage, the variable property_claims reports the number of claims related to material and personal damages when the policy type corresponds to comprehensive coverage, with or without excess (*COMP_E* or *COMP_N*). Similarly, theft_claims,fire_claims and glass_claims capture the number of claims associated with theft, fire and glass coverages, respectively. Complementary coverages are also included through legal_protection_claims, which records the number of legal defence claims and occupants_claims, which reports claims related to bodily injuries sustained by passengers of the insured vehicle.

Regarding claim severity, total_incurred captures the total cost incurred by the insurer as a result of claims, including both paid amounts and outstanding reserves; it is zero in the absence of losses and positive when claims are present. The economic cost of liability claims is reflected in liability_incurred, which aggregates paid and reserved amounts. Consistently, liability_property_incurred and liability_injury_incurred report the incurred cost associated with property damage and bodily injury liability claims, respectively. By construction, the total incurred amount for liability coverage satisfies

liability_incurred=liability_property_incurred+liability_injury_incurred.

For the remaining coverages, property_incurred, theft_incurred, fire_incurred and glass_incurred report the total incurred cost associated with own damage, theft, fire and glass claims, allowing the joint analysis of claim frequency and economic and financial severity. Likewise, legal_protection_incurred and occupants_incurred quantify the incurred costs related to legal defence and occupants coverage, respectively. Thus, the total incurred amount is obtained as the sum of the incurred costs across all coverages as follows:

$$\begin{array}{ccc} total\_incurred & = & liability\_incurred+property\_incurred+theft\_incurred+fire\_incurred\\ & & +\;glass\_incurred+legal\_protection\_incurred+occupants\_incurred. \end{array}$$

Finally, exposure to risk is captured through total_exposure, which represents the time during each calendar year in which the corresponding policy is exposed to risk, measured in policy-years. Its value ranges between 0 and 1, where a value of 1 indicates that the insured remained in the portfolio throughout the entire calendar year, while values below 1 correspond to policies cancelled before year-end or to insured individuals who entered the portfolio during the year. In addition, liability_exposure measures the exposure specifically associated with third-party liability coverage. These exposure variables are essential for the correct normalisation of claims experience and for the computation of actuarial indicators based on rates.

**Fig. 1:**
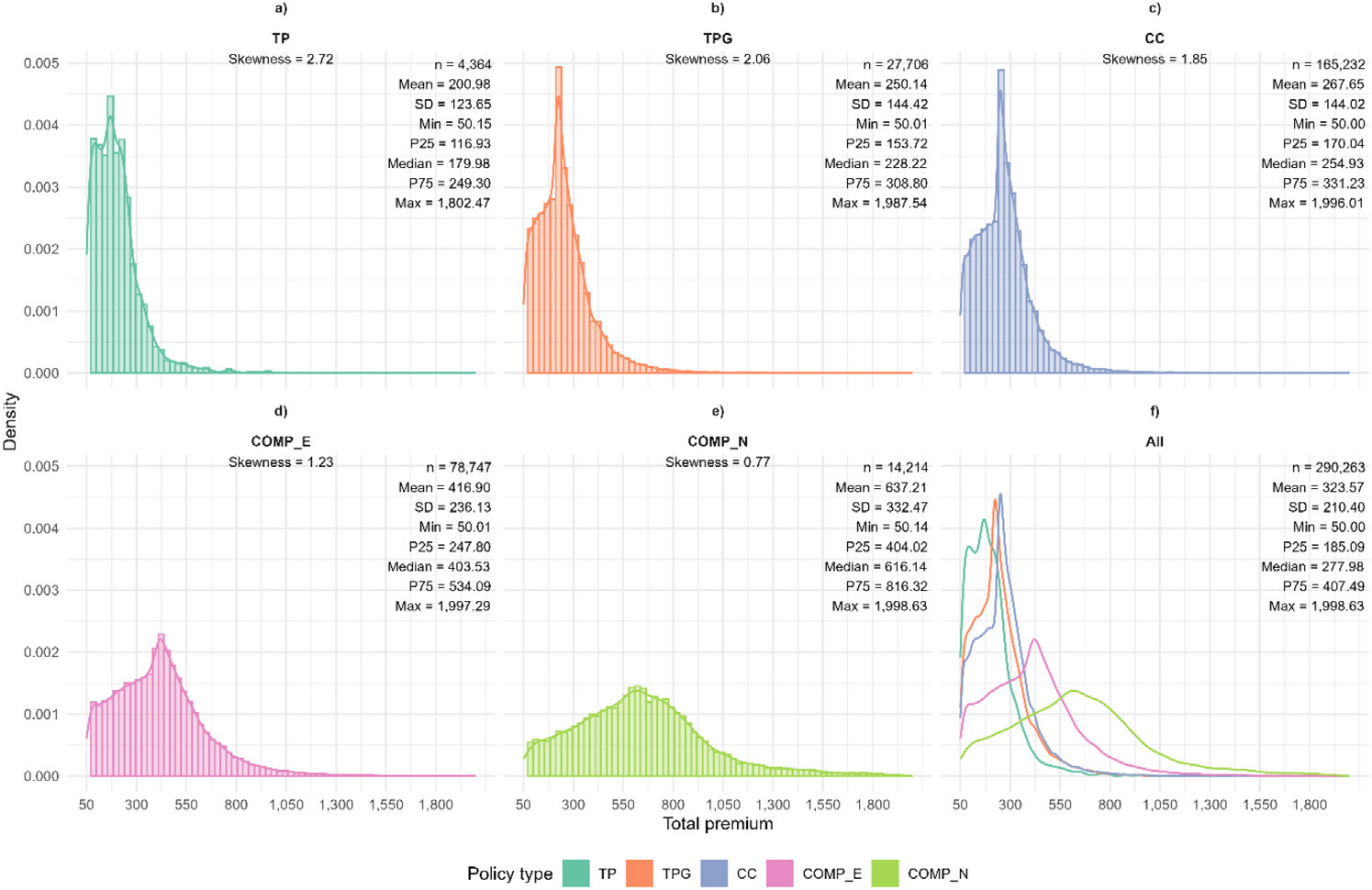
Distribution of premiums by policy type. The “All” panel displays aggregated density functions, while the remaining panels show individual premium distributions for each policy type. Only active policies are included. Note: for graphical representation, only policies with active policy_status (*A*) and total_premium values between €50 and €2000 were considered.

To illustrate an example of the dataset, Fig. 1 presents the distribution of premiums by policy type using histograms and kernel density estimates. The analysis is restricted to observations with positive premiums and values below €2000 (only to provide a better visualization). Fig. 1 is organised into six panels. The last panel (row 2, column 3) displays the aggregated density functions for each policy type, while the remaining first five panels show the corresponding histograms and density estimates separately for each policy type. Descriptive statistics are reported in all panels, with aggregated statistics in the last panel and policy-specific statistics in panels 1 to 5. Additionally, skewness coefficients are reported for the individual distributions. Basic third-party policies (*TP*) exhibit the highest degree of negative skewness, with a coefficient of 2.72, whereas comprehensive policies with and without excess (*COMP_E* and *COMP_N*) display distributions closer to symmetry, with lower skewness values.

## 4 Experimental Design, Materials and Methods

The primary dataset originates from a Spanish motor insurance company and was obtained by the authors in the context of research and data-analysis activities, from an extract of policy and claims records. The microdata were anonymised prior to receipt and further processed by the authors to minimise any residual re-identification risk. It includes detailed information on policy characteristics, vehicle attributes, driver demographics, reported claims and contract dynamics, such as renewals and cancellations (see Table 1, 2, Table 3, Table 4). The dataset covers the period 2022–2024, with the individual policy as the unit of observation, allowing for longitudinal analyses at the contract level within a single insurance entity.

From data reception to the construction of the final dataset, a systematic cleaning and validation process was applied to ensure internal consistency and reproducibility. The initial dataset contained 653,943 rows. However, the insurer recorded every contractual modification (e.g., changes in bank account or driver information) by duplicating the corresponding policy record, differing only in the modified variable. To avoid multiple representations of the same policy-year, only the last available record for each insured and year was retained. After this process, the number of observations was reduced to 354,140.

Categorical variables (policy_type, policy_status, business_type, payment_frequency, bonus_score, fuel_type, municipality_type and circulation_area) were recoded using a standardised nomenclature to correct typographical errors and inconsistent codings. Temporal coherence checks were also performed on time-dependent variables, such as driver_ag*e* and vehicle_age, ensuring consistent evolution across years.

For numeric variables, values were checked to ensure they lay within admissible ranges. In particular, vehicle_value and power_to_weight_ratio were required to be strictly positive; non-compliant values were set to NA. For the variable seats, only strictly positive integer values were retained. Consistency between policy_status and total_exposure was also verified: policies cancelled during the year were required to present exposure values below one, whereas policies active throughout the full year were required to have exposure equal to one.

The variable vehicle_brand was subject to a specific validation and normalisation process to ensure internal consistency. Inconsistencies arising from differences in capitalisation and typographical errors were identified and corrected. As a result, alternative representations of the same brand (e.g., “RENAULT”, “Renault”, or misspelled variants) were unified under a standardised denomination, reducing artificial category fragmentation and improving data quality for subsequent analyses.

Premium-related variables, identified by the term premium in their names, were verified to ensure strictly positive numeric values. Internal consistency checks were performed to confirm that the sum of coverage-level premiums (*liability, property_damage, theft, fire, glass, legal_protection* and *occupants*) matched the reported total_premium. Observations failing this condition were excluded from the computation of total premiums.

Similarly, claim cost variables, identified by the term *incurred*, were verified to ensure non-negative values, as negative incurred amounts lack a direct economic interpretation in the context of this dataset. In the Spanish insurance market, negative incurred values may occasionally arise due to claim reversals or overprovisioning adjustments during the claims-handling process, a practice that is not standard in all international datasets. To maintain comparability and ensure a consistent actuarial interpretation, observations presenting negative incurred values were therefore excluded from the aggregation of *total_incurred*.

Observations failing any of the validation criteria described above were treated as outliers and excluded, while the remaining records were retained for the construction of the final cleaned dataset. To maintain focus on the validated dataset and avoid unnecessary complexity, the raw data are not included in this article. However, they are available from the authors upon reasonable request. The present work therefore concentrates on the structure processing, and validation of the final net dataset.

## Limitations

This dataset presents some limitations that should be acknowledged when conducting empirical analyses. First, information on the distribution channel of the policies (e.g., agents, brokers or bancassurance) is not available. Premium levels may differ across channels due to variations in intermediation costs and commission structures. Therefore, the absence of this variable limits the contextualisation of observed premium heterogeneity. This level of disaggregation is not included due to confidentiality restrictions imposed by the insurer.

Second, claim amounts may be influenced by the institutional framework of the CICOS agreement (Direct Compensation Agreement between insurers) in Spain. Under this system, the insurer of the injured party is responsible for claim assessment and compensation, applying pre-agreed amounts between participating insurers regardless of actual damage. As a result, indemnities may exhibit clustering or systematic patterns that should be considered in claim severity analyses.

Finally, the dataset does not include information on the insured’s sex. Following the ruling of the Court of Justice of the European Union [14], insurers operating in the European Union are prohibited from using gender as a pricing factor. The inclusion of gender could be of interest for descriptive analyses or reserving processes.

## Ethics Statement

The relevant informed consent was obtained by the company from the insured in the moment of contracting the product. Data is offered anonymised.

## CRediT authorship contribution statement

**Priscila Espinosa:** Methodology, Software, Visualization, Resources, Data curation, Investigation, Writing – original draft. **Josep Lledó:** Data curation, Conceptualization, Methodology, Supervision, Visualization, Funding acquisition, Investigation, Writing – review & editing. **David Atance:** Investigation, Visualization, Supervision, Writing – review & editing.

## Data Availability

Mendeley DataA detailed dataset of motor insurance policies with coverage-specific financial information (Original data)

## Appendix Supplementary materials

Image, application 1

Image, application 2

## References

[1] Rodríguez-López G., Serrano A., Martín-Retortillo M., Cazcarro I. (2025). HISTORECO: historical Spanish transition database on climate, geography and economics of the 20th-21st century. Sci. Data.

[2] A.L. Simpson et al. A large annotated medical image dataset for the development and evaluation of segmentation algorithms, 2019, arXiv preprint arXiv:1902.09063. 10.48550/arXiv.1902.09063.

[3] Pérez V., Aybar C., Pavía J.M. (2022). Dataset of the COVID-19 lockdown survey conducted by GIPEyOP in Spain. Data Br..

[4] Pavía J.M. (2022). ei.Datasets: real data sets for assessing ecological inference algorithms. Soc. Sci. Comput. Rev..

[5] Lledó J., Pavía J.M. (2022). Dataset of an actual life-risk insurance portfolio. Data Br..

[6] Lledó J., Espinosa P., Pérez V. (2026). A dataset for health insurance analysis: integrating individual and area-based contextual variables. Sci. Data.

[7] Guillen M., Bolancé C., Frees E., Valdez E.A. (2021). Case study data for joint modeling of insurance claims and lapsation. Data Br..

[8] Yankol-Schalck M. (2022). The value of cross-data set analysis for automobile insurance fraud detection. Res. Int. Bus. Fin..

[9] Segura-Gisbert J., Lledó J., Pavía J.M. (2025). Dataset of an actual motor vehicle insurance portfolio. Eur. Actuar. J..

[10] So B., Boucher J.P., Valdez E.A. (2021). Synthetic dataset generation of driver telematics. Risks.

[11] Boucher J.P., Steven C., Montserrat G. (2017). Exposure as duration and distance in telematics motor insurance using generalized additive models. Risks.

[12] Ayuso M., Montserrat G., Jens P.N. (2019). Improving automobile insurance ratemaking using telematics: incorporating mileage and driver behaviour data. Transportation.

[13] Guillen M., Jens P.N., Pérez-Marín A.M., Elpidorou V. (2020). Can automobile insurance telematics predict the risk of near-miss events?. N. Am. Actuar. J..

[14] Court of Justice of the European Union. (2011). Case C-236/09, association Belge des consommateurs test-Achats and others v. Conseil des ministres (Judgment of 1 March 2011), ECLI:eU:c:2011:100.

